# A Consensus Network of Gene Regulatory Factors in the Human Frontal Lobe

**DOI:** 10.3389/fgene.2016.00031

**Published:** 2016-03-08

**Authors:** Stefano Berto, Alvaro Perdomo-Sabogal, Daniel Gerighausen, Jing Qin, Katja Nowick

**Affiliations:** ^1^Bioinformatics Group, Department of Computer Science, and Interdisciplinary Center for Bioinformatics, University LeipzigLeipzig, Germany; ^2^Paul-Flechsig Institute for Brain Research, University of LeipzigLeipzig, Germany; ^3^Department of Neuroscience, University of Texas Southwestern Medical CenterDallas, TX, USA; ^4^Department of Mathematics and Computer Sciences, University of Southern DenmarkOdense, Denmark; ^5^Institute for Theoretical Chemistry, University of ViennaVienna, Austria

**Keywords:** transcription factor, coexpression network, weighted topological overlap network, consensus network, cognitive abilities, cognitive disorders, prefrontal cortex (PFC)

## Abstract

Cognitive abilities, such as memory, learning, language, problem solving, and planning, involve the frontal lobe and other brain areas. Not much is known yet about the molecular basis of cognitive abilities, but it seems clear that cognitive abilities are determined by the interplay of many genes. One approach for analyzing the genetic networks involved in cognitive functions is to study the coexpression networks of genes with known importance for proper cognitive functions, such as genes that have been associated with cognitive disorders like intellectual disability (ID) or autism spectrum disorders (ASD). Because many of these genes are gene regulatory factors (GRFs) we aimed to provide insights into the gene regulatory networks active in the human frontal lobe. Using genome wide human frontal lobe expression data from 10 independent data sets, we first derived 10 individual coexpression networks for all GRFs including their potential target genes. We observed a high level of variability among these 10 independently derived networks, pointing out that relying on results from a single study can only provide limited biological insights. To instead focus on the most confident information from these 10 networks we developed a method for integrating such independently derived networks into a consensus network. This consensus network revealed robust GRF interactions that are conserved across the frontal lobes of different healthy human individuals. Within this network, we detected a strong central module that is enriched for 166 GRFs known to be involved in brain development and/or cognitive disorders. Interestingly, several hubs of the consensus network encode for GRFs that have not yet been associated with brain functions. Their central role in the network suggests them as excellent new candidates for playing an essential role in the regulatory network of the human frontal lobe, which should be investigated in future studies.

## Introduction

Broadly defined, cognition refers to the biological mechanisms through which animals perceive, learn and memorize information from the environment and decide to act upon them (Shettleworth, 2009). In humans, cognitive processes such as use of language, social behavior, and decision-making have been attributed to the frontal lobe (Duncan et al., 1996; Chayer and Freedman, 2001). However, the actual molecular mechanisms that underlie these morphological changes are still not well understood.

Candidate genes that are involved in the molecular mechanisms of cognition can be identified through biomedical studies on cognitive disorders. For example, causative mutations point to the genes that should in their wild-type variants be important for providing for healthy cognitive abilities. Research on cognitive disorders such as Alzheimer's disease (AD; Bullido et al., 1998), intellectual disability (ID; Kaufman et al., 2010), autism spectrum disorder (ASD; Bailey et al., 1996; Voineagu et al., 2011; Berg and Geschwind, 2012; Ecker et al., 2012), schizophrenia (SZ; Andreasen, 1995), circadian rhythm and bipolar disorder (BD; Akula et al., 2014, 2016; Takahashi, 2015), Parkinson's disease (PD; Polymeropoulos, 2000), and several syndromes or disorders associated with ID or cognitive impairment (SY; Greydanus and Pratt, 2005) has thus already identified several candidate genes involved in cognition. Importantly, these studies also revealed that most cognitive disorders are complex and phenotypically and genetically heterogeneous (Sebat et al., 2007; Tsankova et al., 2007; Voineagu et al., 2011; Weyn-Vanhentenryck et al., 2014), thus creating challenges for studying these disorders.

Transcriptome and network analyses bear great potential for overcoming some of these challenges and uncovering the genetic interactions and molecular mechanisms causing such complex disorders. For example, recent studies have used network approaches to identify coexpressed ASD and ID modules implicated in synaptic development, chromatin remodeling and early transcriptional regulation (Parikshak et al., 2013; Willsey et al., 2013; De Rubeis et al., 2014). However, coexpression networks can have many false positive inferences. One way to reduce the effect of false positives is to calculate weighted topological overlap (wTO) networks (Zhang and Horvath, 2005; Nowick et al., 2009). Another drawback is that most network studies so far have only analyzed data from one dataset. However, it is unclear how variable independently derived networks are and depend, for instance, on the technical platform or on the particular samples/individuals that were used to produce the dataset. We thus analyzed and compared here 10 different transcriptome datasets from individual human frontal lobe samples, which have been produced with different platforms (microarrays and RNA-Seq), and developed a method for integrating the coexpression wTO networks calculated from them into one consensus network of high confidence level.

Several reasons prompted us to especially focus on the role of gene regulatory factors (GRFs) in the consensus network of the frontal lobe. First, because GRFs regulate the expression of many genes, they are expected to be among the most important players in these networks and might provide important insights about the molecular mechanisms taking place in this tissue. Second, primate specific zinc finger genes with a *Krüppel*-associated box (KRAB-ZNFs) are also enriched among the genes expressed during frontal lobe development (Zhang et al., 2011), which leads to the hypothesis that at least some GRFs might contribute to human specific cognitive abilities. Third, we show here that GRFs are enriched among the candidate genes for ID and ASD, thus suggesting an important role of GRFs in the gene regulatory processes and circuitry of such cognitive disorders. Taken together, GRFs are thus good candidates for providing essential information about the molecular mechanisms that set the stage for cognition.

To identify and analyze GRF proteins with potential implications in cognition in more detail, we used our in-house list of all 3315 human GRFs (Perdomo-Sabogal et al., under preparation). This catalog includes information from the most relevant studies in the area of human GRF inventories (see Section Materials and Methods), and includes information about proteins involved in different regulatory mechanisms such as DNA-binding proteins, cofactors that associate with transcription factors, histone and chromatin modifiers, among others. We also performed a comprehensive literature survey and compiled a list of 676 GRFs that are known to be important during human brain development or that have been associated with cognitive disorders. We will refer to this set of 676 GRFs as “Brain-GRFs” (Table S1). Using our high-confidence consensus network we identified here several GRFs, including 166 “Brain-GRFs” that are hubs and thus seem to be important for the gene regulatory processes in the human frontal lobe.

## Materials and methods

### Data sets

The raw and processed data from microarrays and RNA-Seq were downloaded from ArrayExpress (http://www.ebi.ac.uk/arrayexpress/) and Gene Expression Omnibus (http://www.ncbi.nlm.nih.gov/geo/). Microarrays were analyzed using the R programming language and Bioconductor packages (Ihaka and Gentleman, 1996). For the microarrays, we determined gene expression levels (RMA values) and MAS5 detection *p*-value from the probes using the “affy” and “oligo” package, respectively of the platform used (Gautier et al., 2004; Carvalho and Irizarry, 2010). We considered only the probesets significantly detected in at least one individual (*p* < 0.05). Furthermore, for genes represented by more than one expressed probeset, we calculated the mean of the expression values of all its probesets. For the RNA-Seq data, we used published RPKM values when available (BrainSpan). Otherwise, we processed and analyzed the raw data by mapping of the reads using segemehl (Hoffmann et al., 2009) and calculating RPKM values using R programming language and R libraries such as GenomicRanges, GenomicFeatures, and Rsamtools (Lawrence et al., 2013). All the raw data were mapped to the hg19 genome. All expression values were then filtered for RPKM values > 0.5 for 90% of the samples. All samples were used from the following datasets: FrontalVal [GSE25219] (Kang et al., 2011), NeoVal [GSE11512] (Somel et al., 2009), KhatVal [SRA028456] (Somel et al., 2011), and GexVal [GSE22521] (Liu et al., 2012). Only the data from the control individuals were selected from the DisVal [GSE53987], BipRval [GSE53239] (Akula et al., 2014), and BipVal [GSE5388] (Ryan et al., 2006) datasets. From the BrainSpan dataset we selected the samples from the frontal lobe regions and subset them such that individuals with same ages (13 total individuals per dataset) were used.

### Catalog of gene regulatory factor proteins

The GRF catalog we used for building our GRFs consensus network of the human frontal lobe was initially built by Perdomo-Sabogal et al. (under preparation). For this catalog the information for 3315 GRF proteins sourced from the most seminal studies in the area of human GRF inventories (Messina et al., 2004; Vaquerizas et al., 2009; Ravasi et al., 2010; Nowick et al., 2011; Corsinotti et al., 2013; Tripathi et al., 2013; Wingender et al., 2013, 2015) that are associated with gene ontology terms for regulation of transcription, DNA-depending transcription, RNA polymerase II transcription cofactor and co-repressor activity, chromatin binding, modification, remodeling, or silencing, among others, were manually curated.

### Gene sets

The ASD gene list was compiled using the SFARI gene database (09/20/2015, 740 genes; Basu et al., 2009; Banerjee-Basu and Packer, 2010). In the analysis, we included all the 740 genes. In addition, we also calculated the overlap between GRFs and ASD genes with strong association with S category (syndromic) and strong evidence (levels 1–4). ASD modules (asdM12 and asdM16) were obtained from an independent genome-wide expression study that compared ASD with healthy post-mortem brain tissues (Voineagu et al., 2011).

GRFs with association with Parkinson's disease, Alzheimer's disease, and Schizophrenia where filtered according to their significant evidence in more than two GWAS studies (Allen et al., 2008; Bertram, 2009; Jia et al., 2010; Lill et al., 2012). Additional schizophrenia GRFs were derived from independent publication with 108 loci implicated in schizophrenia (Consortium SWGotPG, 2014). ID and FMRP targets genes were collected from independent publications (Inlow and Restifo, 2004; Ropers, 2008; Darnell et al., 2011; van Bokhoven, 2011; Lubs et al., 2012; Consortium SWGotPG, 2014).

Other brain related GRFs were manually selected using web sources such as OMIM and independent databases such as SGZR (Hamosh et al., 2005; Jia et al., 2010). We prioritize GRFs that have evidence on brain functions, synaptic transmission, and brain development.

### wTO calculation

Spearman rank correlations were used to correlate the expression values of the GRF genes with the expression values of all genes, separately in each of the 10 datasets. Note that only expressed genes were considered in each dataset and that the number of expressed GRFs and other genes differs between the datasets. We extracted all significant correlations (*p* < 0.05) for calculating the weighted topological overlap values (ω = *wTO*) between all pairs of expressed GRF genes for each dataset as previously described (Nowick et al., 2009). The calculation is based on a real symmetric matrix A = [*a*_*ij*_], in which *a*_*ij*_ is a real number ranging between −1 to 1 that indicates the correlation coefficient between the *i* -th and *j* -th GRF in the dataset. In particular, we have *a*_*ii*_ = 0. Comparing with the previous method (Zhang and Horvath, 2005), our method incorporates both significant (Spearman rank correlation; *p* < 0.05) positive and negative correlations of two GRFs' correlated gene sets (*u*) described as follow: *a*_*ij*_ ϵ [0, 1] when *a*_*ij*_ ≥ 0 → *a*_*iu*_*a*_*ju*_ ≥ 0 for all *u* and *a*_*ij*_ ϵ [−1, 0] when *a*_*ij*_ ≤ 0 → *a*_*iu*_*a*_*ju*_ ≤ 0 for all *u*. This condition results in a positive wTO value for the GRFs i and j if they are both correlated in the same direction with *u*, while in a negative wTO value if *i* and *j* are correlated with u in the opposite direction.

Inserting the weighted connectivity of a node *i* as:

$$K_{i\;}\text{=}\sum_{i}a_{ij},$$

and the connectivity between i and j as: *C* = *A* * *A*^*T*^, the weighted topological overlap is calculated as:

$$\omega_{ij}=\frac{c_{ij}\;+\;a_{ij}}{min\left(K_{i},K_{j}\right)\text{+' '1-}\left|a_{ij}\right|}$$

To evaluate the reliability of each wTO network, we performed 100 permutations by randomizing the expression values of each individual. This effectively assigned a random expression value to each gene of a particular individual out of all the available gene expression values for that individual. The permutation was done separately for each individual. We then calculated 100 permuted wTO networks for each dataset. We determined the number of links in the empirically derived (“real”) network for multiple wTO cutoffs [0.1:0.6] and compared it to the number of links with the same wTO cutoff in the 100 permuted networks. This method allowed us to determine a *p*-value for how different the empirical networks are from random expectation and to calculate a false positive rate for the links in each network. All empirically derived networks had more links at all tested wTO values compared to the permuted networks, demonstrating that the empirically derived networks are different from random expectation (Table S2D).

### Consensus network construction

To construct the consensus network, we first analyzed the distributions of the wTO values of all GRF-GRF pairs across all datasets using the boxplot.stats function in R (Williamson et al., 1989) to have an overall view of the data sets. Our results show that the distributions of wTO values of the datasets BipRVal, DisVal, and FrontalVal are different from the other datasets (**Figure 2**). Based on these observations, we chose the Wilcoxon rank sum test for our subsequent analysis, since it is a non-parametric test and hence robust against outliers. Thus, we are able to construct the consensus network by taking all the wTO values from all the datasets into consideration. Furthermore, to identify significant GRF-GRF pairs, we performed another Wilcoxon rank sum test with alternative hypothesis greater than |wTO|> 0.3. By applying this test, we avoided potential false positive links due to high variation of wTO values across the datasets. If the result was significant (*p* < 0.05), we considered this GRF-GRF pair as a significant pair. For each of these detected significant GRF-GRF pairs, we then calculated its consensus wTO value as the median of all 10 individual wTO values. Note here, we opted for |wTO|>0.3 as cutoff in the hypothesis, because this was the mean of the cutoffs at which the 10 networks differed from random expectation with *p* < 0.01.

### Network visualization

For network visualization, we used Cytoscape 3.0. Node attributes were used according to our manually curated Brain-GRF list, the Human Proteome map (Kim et al., 2014), and the FMRP targets (Darnell et al., 2011). We included the Cytoscape session (the file is publically available on http://www.nowick-lab.info/?page_id=470) for manual visualization of GRF-GRF interactions as additional file.

### Statistics

For gene set enrichments, *p*-values were calculated with a one-sided Fisher's exact test function in R (alternative = “g,” confidence level = 0.99, simulated *p*-value with 1000 replicates). A one-sided Wilcoxon ranked test was implemented to evaluate the enrichment of the connectivity between species (alternative = “g,” confidence level = 0.99, paired = FALSE). *P*-values for overlaps were calculated with hypergeometric tests using a custom made R script. We retained an independent background (BrainSpan expressed gene = 15585 genes). *P*-values were subsequently adjusted for multiple comparisons using Benjamini-Hochberg FDR procedure. Two-way permutation test of 1000 was performed to validate the overlaps. First we randomized the external gene sets (e.g., ASD genes) by randomly selecting the same number of genes from an independent brain expressed genes list (e.g., BrainSpan gene set) and subsequently calculating the overlap *p*-values with the GRF gene set. The second approach randomized the internal gene sets (e.g., GRF gene set) by randomly selecting the same number of genes as GRFs that were expressed and subsequently calculating the overlap *p*-values. Analysis for RNA-seq, microarray, and correlation filtering were performed using custom made R and SQL scripts. To calculate the correlation and wTO, we developed a Java-based program.

### Enrichment for transcription factor binding sites (TFBS)

For the TFBS enrichment, we focused on the 5421 genes that are expressed in all datasets and correlated with at least one GRF in each of the 10 different datasets. To test whether correlated genes might be target genes of the respective GRF, we performed a ChIP Enrichment Analysis (ChEA) using the ENCODE database and data from Chip-Seq, Chip-Chip, Chip-PET, and DamID experiments (Lachmann et al., 2010). We also performed a TFBS enrichment analysis using the Jolma and JASPAR databases (Jolma et al., 2013; Mathelier et al., 2014). We tested for enrichment of TFBSs included in those databases within the 2 kb upstream region of the 5421 genes using CentriMo (default parameters) implemented in the MEME suite (Bailey et al., 2009; Bailey and Machanick, 2012). As background, we used the 2 kb upstream regions of the remaining protein coding genes and CpG islands.

### Protein–protein-interactions enrichment

Protein–Protein-Interactions (PPIs) were compiled from BioGRID and InWeb using the method described in Parikshak et al. (2013). We used the set of 5421 genes commonly expressed in all 10 datasets. Then we determined the GRF-gene pairs that were called to interact as proteins according to BioGRID and InWeb (Rossin et al., 2011; Chatr-Aryamontri et al., 2013). GRF-gene pairs that were present in each of the 10 datasets and were indicated to interact as proteins were then combined to a consensus PPI network. Fisher's exact test was used for testing the enrichment of PPI in Brain-GRFs and other GRFs.

### GO enrichment

For the GO enrichment analysis in the consensus network, we first ranked the genes of each dataset according to the number of GRFs they were correlated with. Then we summed up the ranks across the 10 datasets. The ranked list of the sums of the ranks was used as input for the Wilcoxon test implemented in FUNC (Prüfer et al., 2007) for the GO enrichment analysis. This method allowed us to understand the relative importance of a gene in each dataset according to the rank position. We next summarized the ranks across the 10 datasets, thus obtaining a general rank (rank-sum). The GO enrichment test was performed using FUNC (Prüfer et al., 2007). We used a Wilcoxon rank-based test for GO enrichment among the genes with highest rank-sums. For the GO analyses we only analyzed GO groups with at least 20 genes per group. We report GO groups with enrichment with *p* < 0.01 before and after refinement.

For the analysis of GO enrichment within each individual network among genes correlated with the selected Brain-GRF hubs we collected for each hub its correlated genes in all the 10 datasets. The remaining set of expressed genes was used as background set. We used the hypergeometric test implemented in FUNC for the GO enrichment analysis considering only GO groups with at least 20 genes per group. We report GO groups with enrichment with *p* < 0.01 before and after refinement. Finally, we summarized the 10 lists of significant GO categories into one single list, thus removing duplicated GO categories. We also parsed the analyzed GO categories into a list of developmental categories using CateGOrizer (Hu et al., 2008).

## Results

### Gene regulatory factors involved in brain development and cognitive disorders

**Figure 1:**
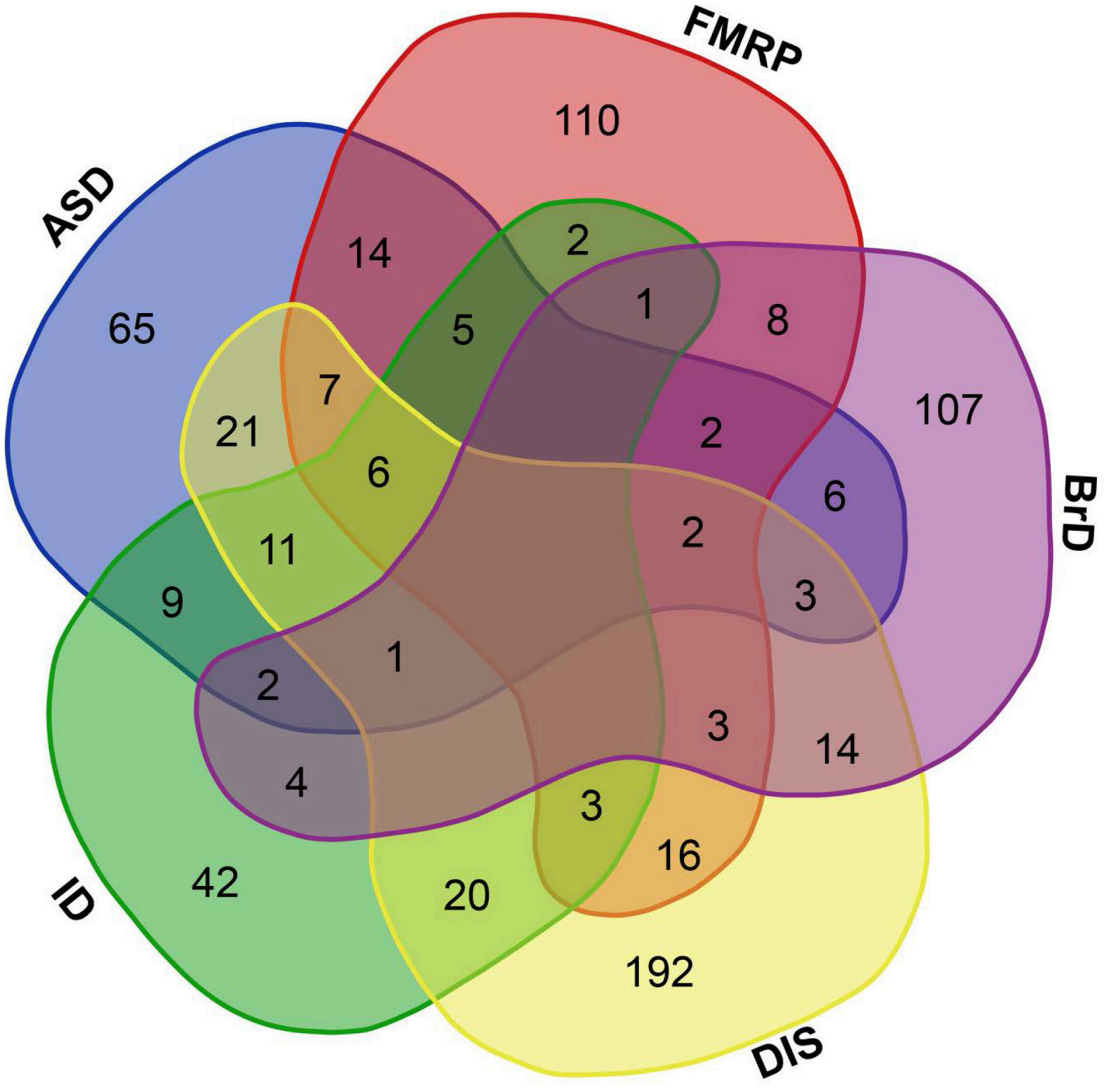
**Brain-GRFs association**. Overlap between GRFs implicated in autism (ASD) or intellectual disability (ID), GRFs that are FMRP targets (FMRP), GRFs involved in brain development and functions (BrD), and GRFs implicated in syndromes or disorders (DIS). Empty space represents no overlap between sets. The overlap shows the commonalities of GRFs implicated in multiple disorders and syndromes.

Within this list of human GRFs we identified 676 GRFs that are involved in cognitive functions, brain development, and disorders by using different sources (see Materials and Methods; Figure 1 and Table S1). A prevalence of genes coding for GRFs among genes associated with some cognitive disorders has been observed before (Hong et al., 2005; West and Greenberg, 2011; Parikshak et al., 2013; De Rubeis et al., 2014; Nord et al., 2015). We here tested if this observation represents a significant overrepresentation of GRF genes among genes implicated in cognitive disorders. Among the 401 genes implicated in ID, we identified 106 genes coding for GRFs, which represents a highly significant enrichment of GRFs among all ID genes (hypergeometric test, *p* = 2.03 × 10^−07^). The SFARI database (Basu et al., 2009; Banerjee-Basu and Packer, 2010) currently contains 740 genes implicated in autism. Among those, 297 genes show strong evidence of ASD association. We identified 154 GRFs among the 740 genes (78 among the 297 ASD genes with strong association), which demonstrates that there is also a highly significant overrepresentation of GRFs among genes associated with autism (hypergeometric test, *p* = 0.0001). We further investigated whether GRFs are enriched among the target genes of the Fragile-X Mental Retardation Protein (FMRP). This protein was previously shown to play an important role in ASD-pathways by exerting translational regulation during human brain development (Darnell et al., 2011). Among the set of 842 FMRP target genes predicted by HITs-CLIP, we identified 179 GRF genes revealing a significant overrepresentation of GRF genes (hypergeometric test, *p* = 0.0001). In addition, GRFs are also significantly enriched for genes highly expressed in neurons (hypergeometric test, *p* < 0.001) and astrocytes (hypergeometric test, *p* < 0.05) compared with other brain cell-type expressed genes (Zhang et al., 2014).

Taken together, these findings show that GRF genes are enriched among candidate genes for cognitive disorders and cell important for brain functions, metabolism, and structure. Therefore, they are likely to be good candidates for providing essential information about the molecular processes involved in the organization and functioning of neural circuits that support healthy cognitive abilities.

### A consensus network of high confidence

To investigate the roles of all GRFs in the frontal lobe, we analyzed 10 genome-wide expression datasets comprised of frontal lobe samples from individuals of different ages and obtained with different techniques (Table 1). We first analyzed each dataset independently to investigate the consistency of the coexpression networks derived from these independent datasets.

**Table 1:** **Platforms description**.

**Dataset**	**Names**	**Assession number**	**Sample**	**Type**	**Permutation (|wTO|)**
BipRVal	*Bipolar RNA-seq Values*	GSE53239	11	Adult	>0.39
BipVal	*Bipolar Microarray Values*	GSE5388	31	Adult	>0.24
DfcVal	*DFC RNA-seq Values*	BrainSpan	13	Developmental	>0.36
DisVal	*Disorder Microarray Values*	GSE53987	19	Adult	>0.30
FrontVal	*Frontal Pole Microarray Values*	GSE25219	348	Developmental	>0.10
GexVal	*Gene Expression Microarray Values*	GSE22521	25	Developmental	>0.25
KhatVal	*RNA-seq Values from a Khaitovich study*	SRA028456	12	Developmental	>0.36
NeoVal	*Neoteny Microarray Values*	GSE11512	44	Developmental	>0.19
OfcVal	*OFC RNA-seq Values*	BrainSpan	13	Developmental	>0.37
VfcVal	*VFC RNA-seq Values*	BrainSpan	13	Developmental	>0.37

*We used multiple platforms to uncover the GRF—GRF interactions*.

*The first column represents the chosen name of each dataset*.

*The second column showed complete name of the dataset, the used platform, and Values as indication for the wTO calculation*.

*The third column contains the accession numbers of each dataset*.

*The fourth column indicates the number of samples used for the analysis*.

*The fifth column indicates the type of dataset*.

*The sixth column shows the |wTO| cutoff at which the dataset has significantly more links in the empirical network than in the permuted ones*.

*For BipRval, BipVal, DisVal, we used only the control samples consisting of healthy individuals (see Materials and methods)*.

Specifically, from each dataset, we constructed a weighted topological overlap (wTO) network taking into account all expressed GRFs and their coexpressed genes (Nowick et al., 2009). For constructing this wTO network, we first identified all genes that are significantly correlated in expression (i.e., coexpressed) with a particular GRF. These genes include putative target genes and genes coding for interaction partners of that GRF. The wTO of a pair of GRFs then represents the commonality of these two GRFs in their sets of coexpressed genes. Because GRFs can function as activators or repressors of gene expression, we take into account the sign of the correlation when calculating the wTO. Pairs of GRFs with |wTO|values above a certain cutoff are connected by a link in the wTO network visualization (see Materials and Methods).

**Figure 2:**
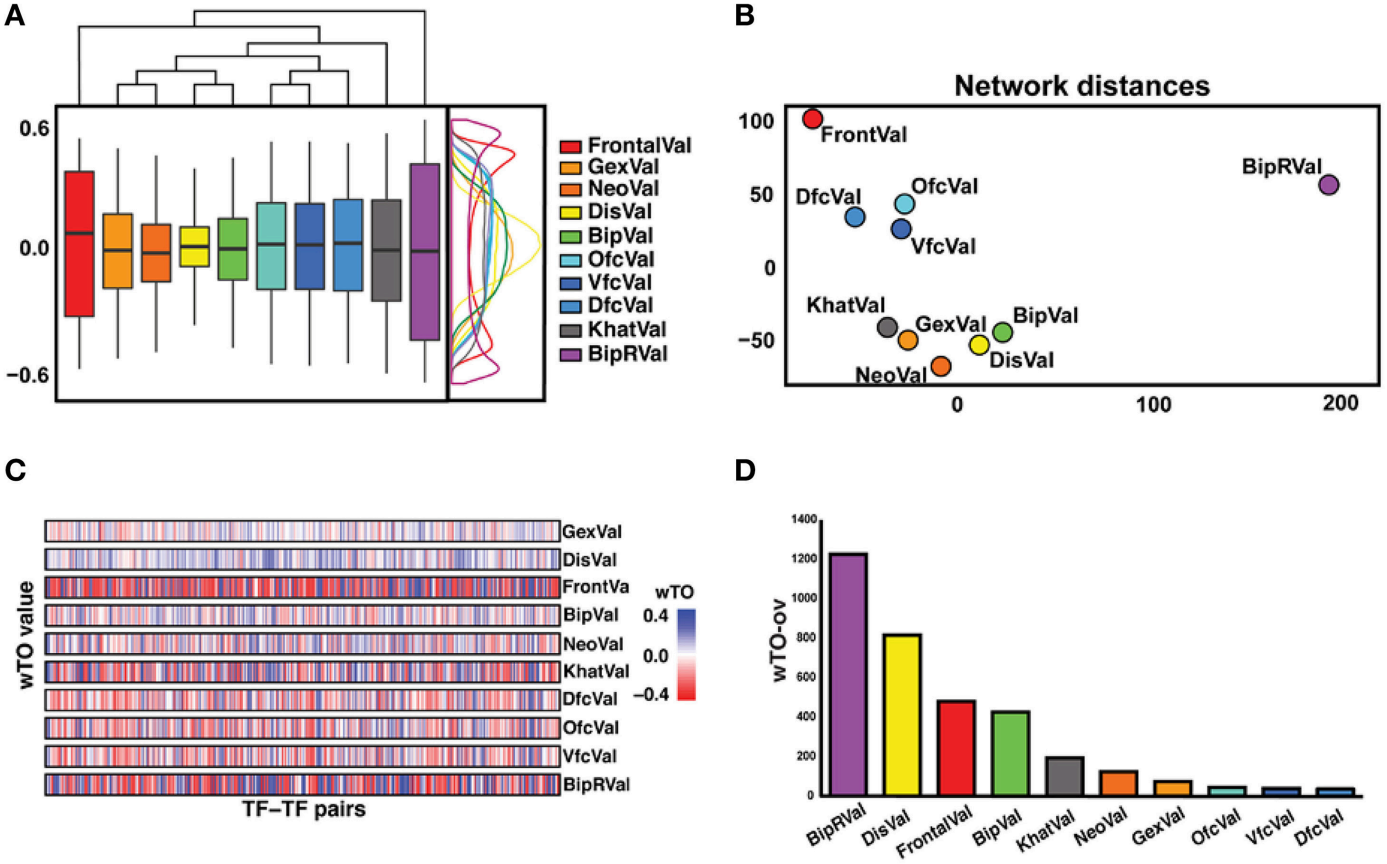
**Overview of differences and similarities between datasets. (A)** Representation of the distribution of the wTO values of the 10 datasets. On the right side, a wTO density plot. On the top, a clustering map of the datasets showing FrontalVal and BipRVal as outliers compared with the remaining datasets. **(B)** Two-dimensional scaling plot in which the circles represent the datasets used in this study. The BipRVal dataset is the most different dataset compared to the other datasets. The three BrainSpan datasets (DfcVal, OfcVal, VfcVal) cluster together. The microarray datasets (GexVal, NeoVal, DisVal, BipVal) showed a consistent clustering with one additional RNA-seq dataset (KhatVal). FrontalVal is not clustering with any of the other microarray or RNA-Seq datasets. This clustering suggests that the wTO networks do not simply cluster according to experimental platforms. **(C)** Overall stripe chart of the wTO values across the 10 datasets. Red represents negative wTO values whereas blue represents positive wTO values. As also seen in Figure 2A, FrontalVal and BipRVal wTO values differ most from the other datasets. **(D)** Barplot representing the numbers of detected wTO outlier values (wTO-ov) per dataset. BipRVal contained the highest number of outliers underlining it as being the most distant dataset.

Even though each network is supported to significantly differ from random expectation, we noted differences between the 10 networks, for instance, in the distribution of the wTO values and when comparing the wTO values for particular links between the datasets (Figures 2A,B). The differences between the networks can probably be explained by biological variation between individuals, but also by technical variations such as in RNA extraction methods, RIN values, and RNA library preparation procedures. We observed that the dataset BipRVal differs the most from the other datasets by having the highest number of wTO outliers, followed by datasets DisVal and FrontalVal (Figures 2B,C). All in all, we found that merely 19% (287930) of all links between GRFs are present in all 10 wTO networks. Given such variation between the networks, we think it is dangerous to rely on only one dataset when making inferences about biological relationships. Instead, multiple datasets should be combined to alleviate the dependence of the results on a particular set of individuals, developmental time points, different RNA library preparations, and gene expression measurement platforms and to focus on the most consistently observed links.

**Figure 3:**
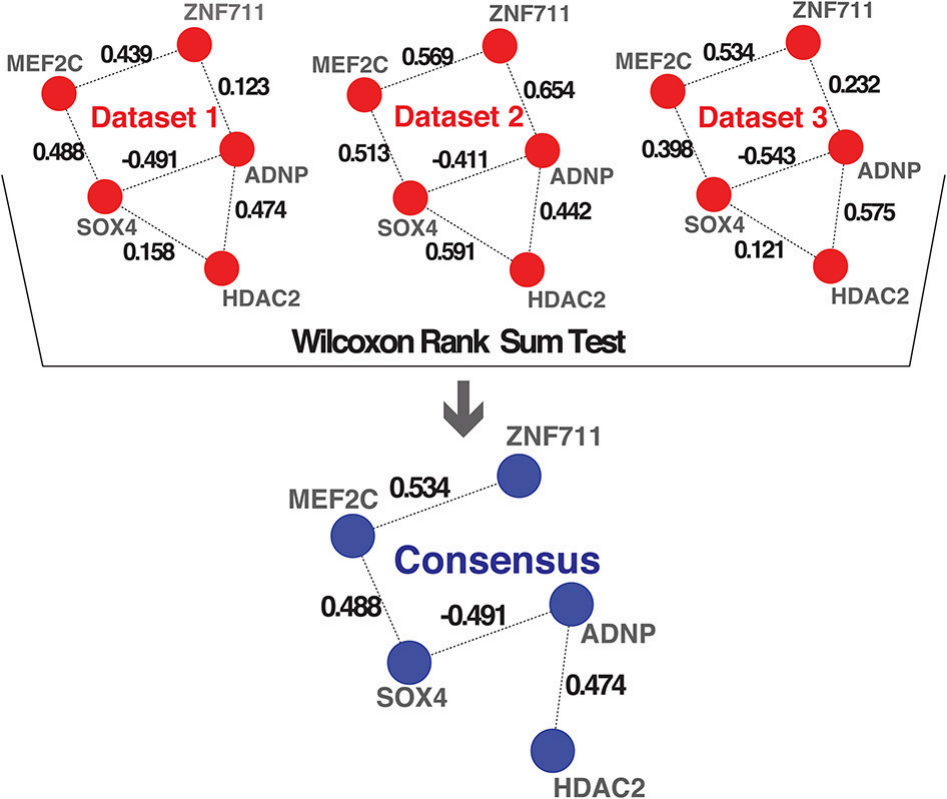
**Consensus method**. Schematic representation of the method we implemented for combining multiple networks into a consensus network. The examples shown in the first part highlight hypothetical interactions present in three independent datasets. The numbers on the links represent the wTO values calculated using our method. We performed a Wilcoxon rank sum test to statistically determine which links had wTO values that were significantly higher than a chosen cutoff (|wTO|> 0.3) across all datasets. The blue network represents the consensus network containing only these significant links. The numbers shown at the links of the consensus network are the median wTO values calculated from the respective links in the 10 datasets. The links that not full-filled our statistical criteria due to high variation between dataset and cutoff trimming were consequently excluded.

**Figure 4:**
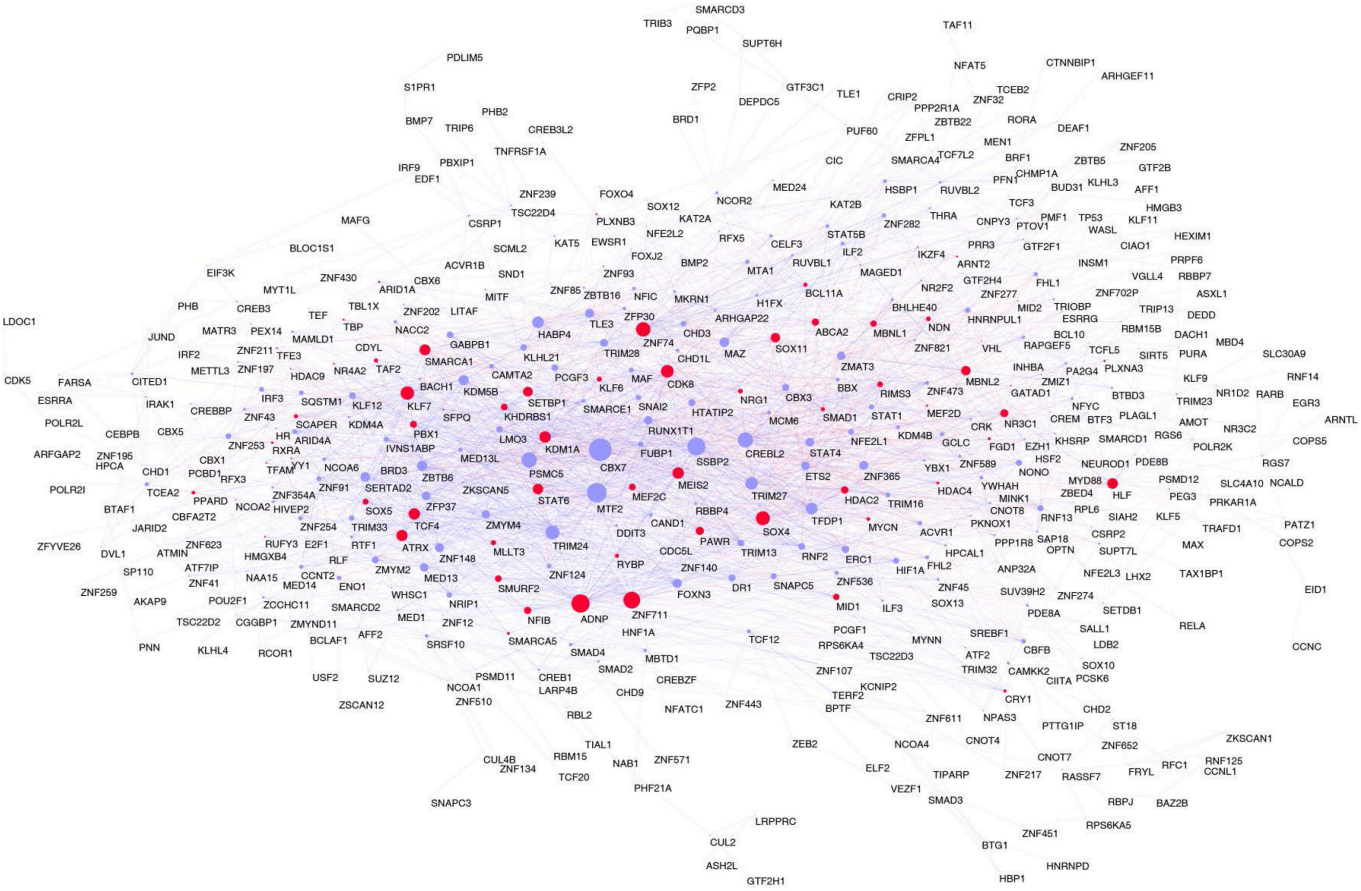
**Consensus network**. In red Brain-GRFs; in blue all other GRFs. Node size is proportional to the number of links. Links with positive wTO values are in blue and links negative wTO values are shown in red.

To combine the 10 independently derived networks into a consensus network with higher confidence, we considered them as biological replicates. We evaluated for each GRF—GRF pair, whether the distribution of strengths of their links across the 0 datasets is significantly higher than a chosen cutoff (Wilcoxon rank sum test, *p* < 0.05; Figure 3 and see Materials and Methods). If so, the link was included into our consensus network. The resulting consensus network for |wTO|>0.3 consists of 2516 links (Figure 4 and Tables S2A,B). This method allowed us to pinpoint the links with the strongest consistency across multiple networks. To determine the final weight of the links in the consensus network, we calculated the median of all wTO values for the respective GRF—GRF pair.

### Brain-GRF genes are often hubs and highly interconnected in the frontal lobe consensus network

Focusing on the most consistent links as determined by our consensus network, we next analyzed how the known Brain-GRFs are integrated into this consensus network. Of the total of 676 Brain-GRFs, 166 are present in the consensus network. Interestingly, this represents a significant enrichment of Brain-GRFs among the 498 GRFs of the consensus network (Fisher exact test, *p* = 1.79 × 10^−11^, Odd Ratio = 2.2). Remarkably, the group of Brain-GRFs has a higher connectivity (number of links) compared to other GRFs in the consensus network (Wilcoxon rank sum test, *p* = 0.015). Those finding suggests that known Brain-GRFs have stronger and more consistent functional relationships amongst each other than other GRFs in the frontal lobe.

To confirm the transcriptional pathways suggested by our consensus network, we examined whether there is enrichment of the GRF binding sites in the regulatory sequences of the 5421 genes that are correlated with at least one of the 498 GRFs of the consensus network (Table S2C). To this end, we first performed a ChIP enrichment analysis (ChEA) using the updated ENCODE database and a manually curated list of target genes uncovered by ChIP-Seq, Chip-chip, ChIP-PET, and DamID from multiple studies (Lachmann et al., 2010). We found that the TFBS of 55 GRFs in the consensus network are significantly enriched among the regulatory sequences of the 5421 genes (*p* < 0.05 after Benjamini-Hochberg correction). Among those 55 GRFs, we found, for instance, *HDAC2* involved in synaptic plasticity and neural circuits (Guan et al., 2009), *ATF2* linked to neuronal apoptosis and cell migration (Yuan et al., 2009), and *CHD2* implicated in ASD and epilepsy (Rauch et al., 2012; Table S3A). Secondly, using the Jaspar and Jolma databases (Jolma et al., 2013; Mathelier et al., 2014), we found an enrichment of binding sites for 34 additional GRFs of the consensus network within the 2 kb region upstream of the transcription start site of the 5421 genes (Fisher exact test, *p* < 0.05 after Benjamini-Hochberg correction; Jolma et al., 2013; Mathelier et al., 2014). Here, we found enrichment for binding sites of *ARNTL*, important for circadian rhythm associated with BD (Nievergelt et al., 2006), *MEF2D*, involved in neuronal differentiation and PD (Yang et al., 2009), and *MEF2C*, involved in ASD, ID, and epilepsy (Novara et al., 2010) among others (Table S3B).

Coexpressed genes can also indicate protein interaction partners. Thus, we next examined protein—protein interactions (PPI) among the 498 GRFs and the 5421 correlated genes utilizing the annotations from BioGRID (Stark et al., 2006) and InWeb. We found that correlated GRF-gene pairs were significantly enriched within the PPI interactions (Fisher exact test, *p* = 2.2 × 10^−6^, Odd Ratio > 3), thus providing an additional confirmation of the potential functional interactions between GRFs and their correlated genes (Table S4).

In addition to the Brain-GRF enrichment, we examined the overlap between our consensus network with two coexpression modules, asdM12 and asdM16, that have been implicated in ASD previously (Voineagu et al., 2011). Remarkably, the consensus network overlaps significantly with the asdM12 module that is associated with synaptic development and dysregulated in ASD brains (hypergeometric test, *p* = 0.045). This result suggests that functional relationship of the GRFs in our consensus network plays a role in ASD.

**Figure 5:**
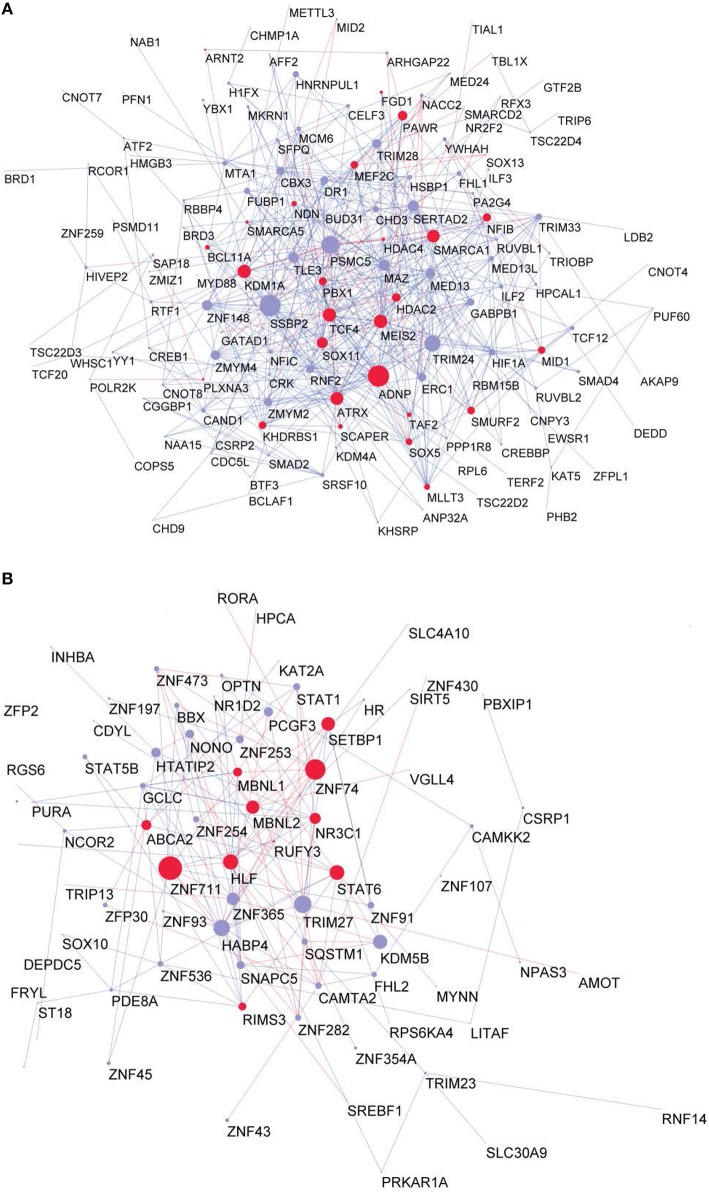
**Proteome GRF modules with red nodes representing the Brain-GRFs whereas in blue the other GRFs**. Links with positive wTO values are in blue and links negative wTO values are shown in red. **(A)** Fetal module. **(B)** Adult module. Brain-GRFs are significantly enriched in the fetal module showing higher connectivity compared with the other GRFs.

To investigate whether the GRFs are also highly expressed at protein level in a fetal or adult brain, we superimposed our consensus network with a proteome map of the human brain at different stages, which was derived using mass-spectrometry proteomics (Kim et al., 2014). This strategy allowed us to understand the potential roles of the GRFs in the period of brain development and circuitry formation compared with an adult brain. Interestingly, overall the GRFs of our consensus network have higher expression and significantly more links in the fetal module compared to the adult module (Wilcoxon rank sum test, *p* = 0.006). The known Brain-GRFs are specifically enriched in the fetal module (Fisher exact test, *p* = 0.03, OR = 1.5) with generally higher number of links in comparison to other GRFs (Wilcoxon rank sum test, *p* = 0.002; Figures 5A,B).

**Figure 6:**
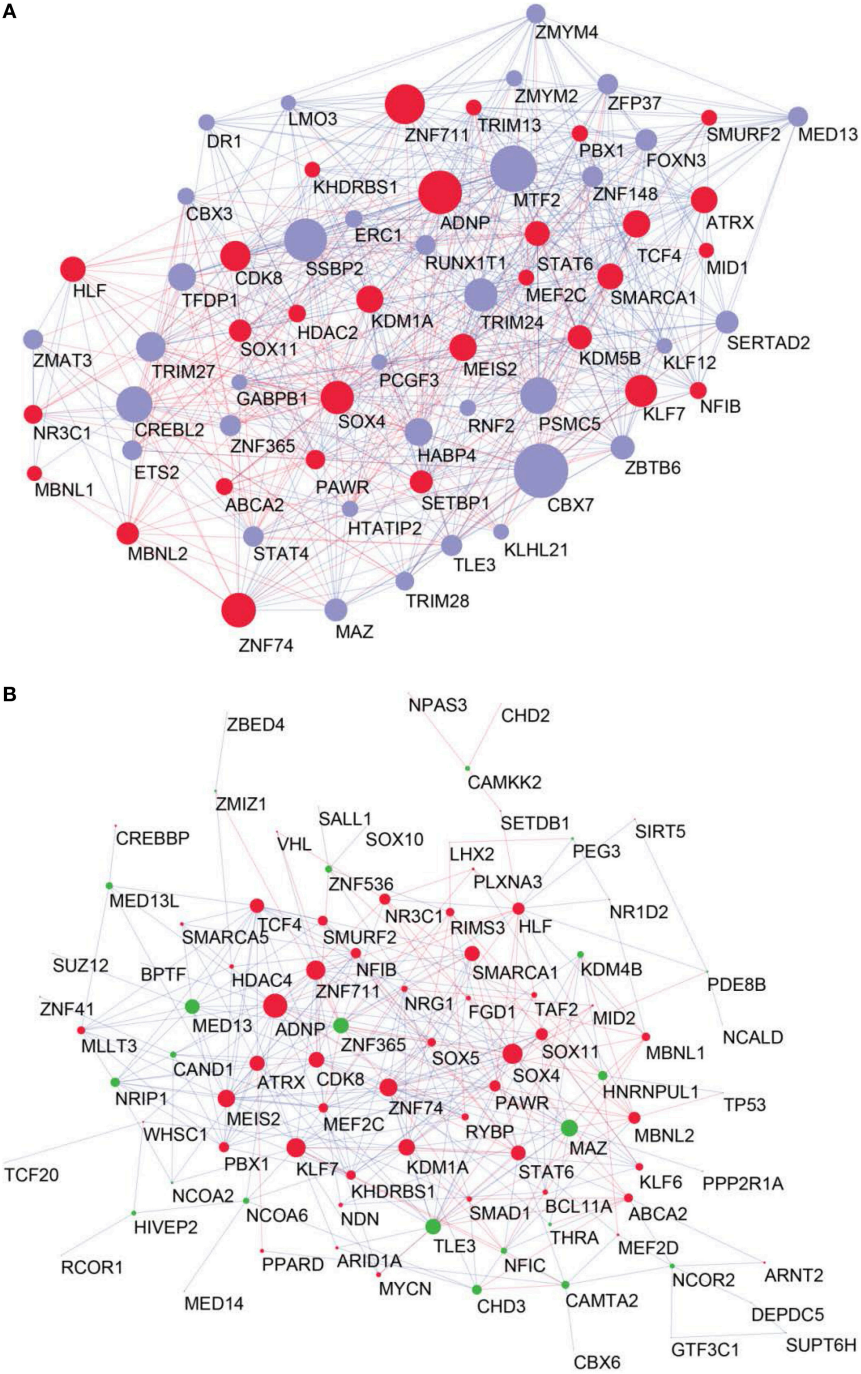
**High confident consensus network and proteomics networks. (A)** Representation of the frontal lobe consensus network. Shown are the most highly connected hubs (degree > 25). Red nodes highlight Brain-GRFs, while blue nodes represent all other GRFs. The size of a node is proportional to its number of links: bigger nodes represent hubs in the network. Links with positive wTO values are in blue and links with negative wTO values are shown in red. **(B)** Brain-GRFs and FMRP targets module. Red nodes highlight the Brain-GRFs, while the green nodes highlight GRFs that are FMRP targets. The size of the nodes is proportional to their number of links.

**Figure 7:**
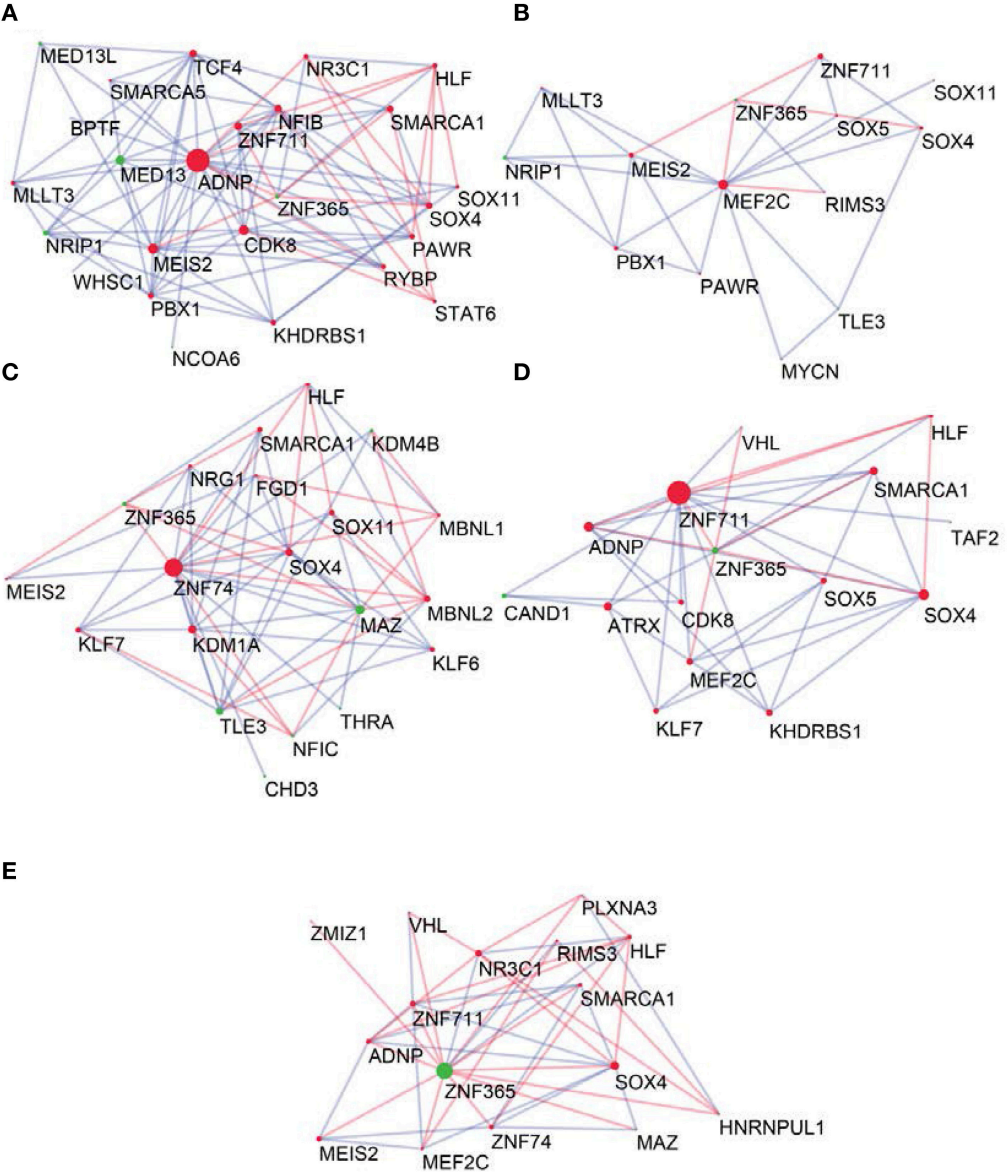
**Neighbors of hub Brain-GRFs and their strongly connected partners. (A)** ADNP module, **(B)** MEF2C module, **(C)** ZNF74 module, **(D)** ZNF711 module, and **(E)** ZNF365 module. Red nodes highlight Brain-GRFs whereas green nodes represent FMRP targets. Links with positive wTO values are in blue and links negative wTO values are shown in red. Each hub Brain-GRFs is interestingly associated with other known Brain-GRFs highlighting potential interactions and common pathways.

To determine the most important GRFs in the consensus network of the human frontal pole, we determined the GRFs with the highest numbers of links (Figures 6A,B). Examples of such hubs include *ADNP, ZFN711, ZNF74*, and *SOX4*, which are all Brain-GRFs. Interestingly, those Brain-GRFs are also strongly interconnected with other Brain-GRFs (e.g., *MEF2C, PBX1, SMARCA1*, an *SOX11*) and GRFs that are FMRP-targets (e.g., *KDM4B, MED13, NRIP1*, and *ZNF365*), suggesting a high functional interrelationship between various Brain-GRFs (Figure 7). Of note, in addition to the Brain-GRFs, the consensus network also contained hubs that yet are not implicated in brain functions or disorders. For example we detected GRFs important for embryogenesis (e.g., *CBX7, TFDP1*, and *TLE3*; Dehni et al., 1995; Morey et al., 2013; Laing et al., 2015) and energy metabolism (e.g., *PSMC5* and *SERTAD2*; Hoyle et al., 1997; Liew et al., 2013). Due to their strong connectivity to known Brain-GRFs in the consensus network, it seems likely that also these GRFs play an important role in the human frontal lobe circuitries. Taken together, our results suggest GRFs that are important for shaping the transcriptional circuitry of the human frontal lobe, including novel candidates for experimental validation of their roles at brain level and potential association with cognitive disorders.

**Figure 8:**
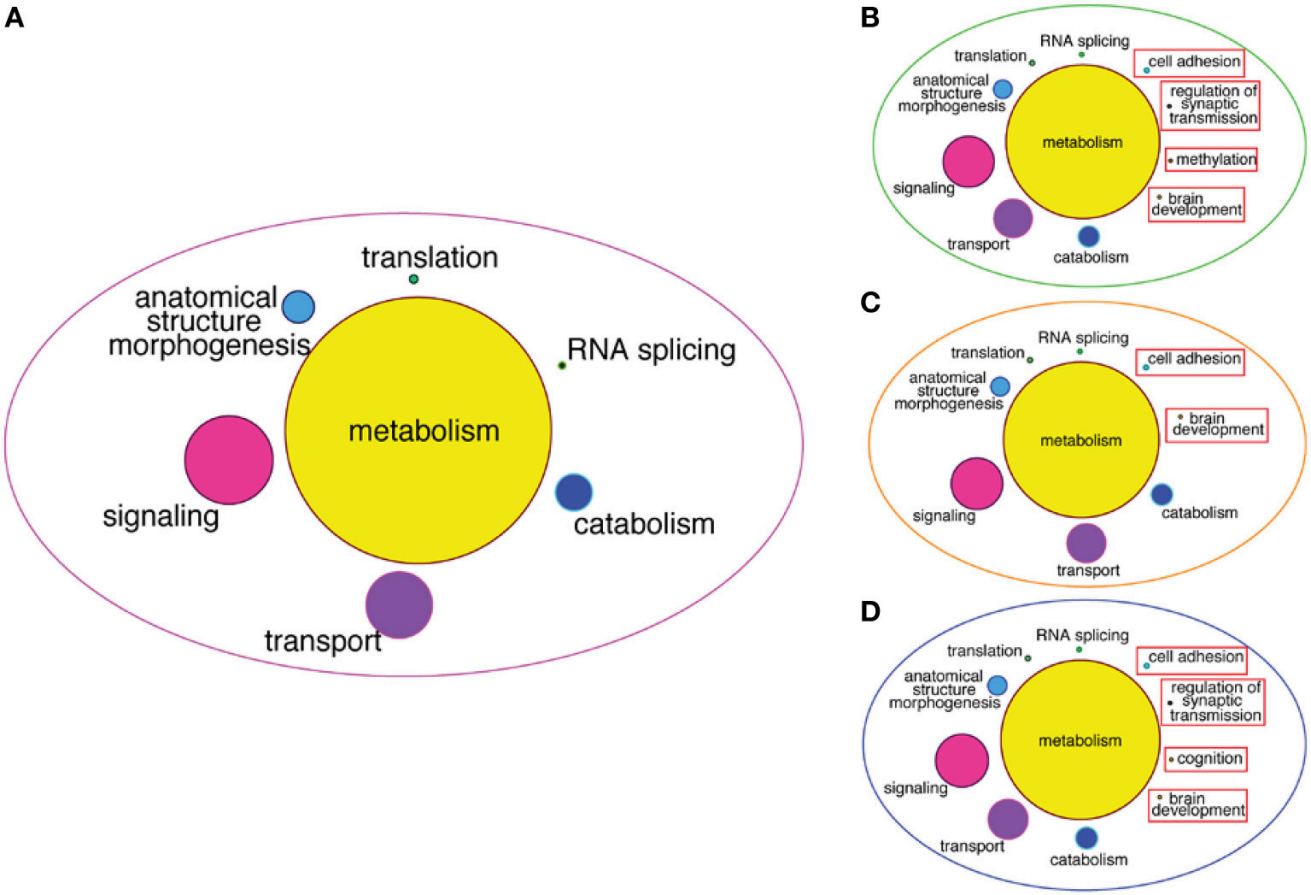
**GO enrichment among correlated genes of the consensus network and of Brain-GRF hubs. (A)** GO categories that are enriched among the correlated genes of the GRFs of the consensus network. The categories for metabolism represent 46% of the enrichment. **(B–D)** GO categories enriched among the correlated genes of three selected Brain-GRF hubs of the consensus network (ADNP, ZNF711, and ZNF74, respectively). Interestingly, Brain-GRFs showed specific enrichment for categories involved in cognition and brain development.

To infer more about the functions of the GRFs in the consensus network, we performed a Gene Ontology (GO) enrichment analysis among the genes correlated with the GRFs (see Materials and Methods). We found significant enrichment for genes involved in metabolism, signaling, transport, translation, and RNA splicing (Figure 8A). We also specifically tested for GO enrichment of the genes correlated with three Brain-GRFs that are the strongest hubs in the consensus network: *ADNP, ZNF711*, and *ZNF74* (see Materials and Methods). Overall, we found similar GO groups enriched for these hubs like we did for the consensus network as a whole. However, there were also hub-specifically enriched GO categories such as brain development, methylation, and regulation of synaptic transmission, which suggests a specific role of these three GRFs in the regulation of genes important for these particular brain functions (Figures 8B–D; Table S5).

## Discussion

Comprehending the characteristic complexity of cognitive disorders, such as ASD and ID, still represents a challenge in neurosciences. An important step toward understanding this complexity is to elucidate the molecular networks of healthy human brains. In this study, we specifically compiled a set of 676 “Brain-GRF” genes implicated in brain development and cognitive disorders and analyzed their co-expression networks to gain first insights into which gene regulatory pathways these genes may be involved in in the frontal lobe of healthy individuals. Importantly, we discovered that networks derived from independent studies differ considerably from each other, highlighting a potential danger of relying on just one dataset. After combining these independent networks into a consensus network containing the links that are the most conserved across them, we were able to identify robust relationships between GRFs in the coexpression network of the frontal lobe of healthy human individuals. We further discovered that, while some hubs in the consensus network are known “Brain-GRF” genes, others have not been linked to functions in the brain before.

The function of most GRFs is still only insufficiently characterized. However, insights into the functions and interactions of our human frontal lobe consensus network can be gained from the expression patterns of the GRFs, the GO enrichment of the genes correlated with the GRFs, and disorders the GRFs have been associated with. Many hubs of the consensus network are also expressed in tissues other than brain. However, we observed that a considerable number of them (115 in total), for example *ZNF711, ADNP, MEF2C, SOX11*, and *CBX7*, have higher expression in mouse neurons than in other brain cells, such as glia, astrocytes, oligodendrocytes, myelinating oligodendrocytes, and endothelial cells (Zhang et al., 2014), suggesting that they have an essential role in neurons. In addition, we also discovered that the GRFs of our network play dominant roles in the fetal proteome module, (Kim et al., 2014) supporting the reasoning that these GRFs might regulate important processes during brain development such as forming the necessary brain structures for proper brain functions, including cognitive functions. Despite being ubiquitously expressed, it is plausible that some GRFs might only be hubs in the frontal lobe, a possibility that needs to be investigated further when data becomes available.

Our GO analysis revealed that the hub GRFs of the frontal lobe consensus network are likely to regulate genes involved in splicing, translation, metabolism, signaling, and synaptic transmission in the frontal lobe. Interestingly, these GO categories seem to be important for several brain functions. For instance, translational mechanisms have been shown to play a role in the mechanisms of memory formation and synaptic plasticity (Richter and Klann, 2009) and RNA splicing mechanisms have been implicated in neuronal development (Li et al., 2007; Weyn-Vanhentenryck et al., 2014). Genes involved in metabolism might be important to provide the brain with the necessary energy for its functions. Signaling and synaptic transmission are important for the communication between neurons and relevant to allow for cognitive abilities. We thus suggest the interactions of the GRFs in the frontal lobe network are critically underlying the regulatory processes that allow for these vital brain functions.

We found a significant enrichment of known Brain-GRFs, including GRFs implicated in ASD, ID, or SY in our consensus network, indicating that it forms the basis for setting the stage for healthy cognitive abilities. For instance, the three strongest hubs are *ZNF711*, associated with ID (Tarpey et al., 2009), *ADNP*, involved in ID and ASD (Helsmoortel et al., 2014; Iossifov et al., 2014), and *ZNF74*, involved in ID and SY (Ravassard et al., 1999). Being in these central network positions presumably renders them to risk genes that increase the likelihood for developing brain disorders. We speculate that interaction between *ZNF711* and *ZNF74* reflect biological pathways that might be important for intellectual abilities. In line with this potential, genes correlated with *ZNF711* and *ZNF74* are enriched for functions such as axon development, brain development and regulation of synaptic transmission, which are likely important for the development and maintenance of healthy cognitive skills. Another hub in our GRF consensus network is *MEF2C*, a GRF that is important for synaptic plasticity and has been implicated in ASD (Ebert and Greenberg, 2013). *MEF2C* is also strongly associated with other Brain-GRFs such as *ZNF711, SOX11*, and *SOX5*, defining a strongly interconnected module of GRFs involved in regulatory pathways that might control cognitive functions (Uwanogho et al., 1995; Jankowski et al., 2006; Tarpey et al., 2009; Schanze et al., 2013). Our analysis highlighted also hubs that are targeted by FMRP, pointing to pathways that might be (dys)regulated at the post-transcriptional level. For instance, *CREBBP*, a GRF associated with ASD and ID (Barnby et al., 2005), *HDAC4*, implicated in ID and ASD (Pinto et al., 2014), *ZNF365*, which has been discovered in a module strongly associated with ASD in a brain expression study (Voineagu et al., 2011), and *KDM5B* and *KDM4B*, recently implicated in ASD using another weighted network approach (TADA; De Rubeis et al., 2014; Iossifov et al., 2014). *CREB* transcription factors and *HDAC4* are further known to regulate synaptic plasticity and memory formation (Silva et al., 1998; Hardingham et al., 2001; Vecsey et al., 2007; Thomson et al., 2008; Kim et al., 2012; Sando et al., 2012). These observations lead us to speculate that Brain-GRFs are strongly dependent on each other by sharing functional pathways and target genes. Further experimental studies are needed to identify shared targets of these and other GRFs to confirm their role in human frontal lobe functions and disorders.

Supporting our speculation that Brain-GRFs depend on each other, we found that Brain-GRFs have significantly more links than other GRFs and are strongly interconnected in the human frontal lobe network. Importantly, in addition to 30 known Brain-GRFs that are hubs, we identified further 36 GRF genes that are hubs in the frontal lobe consensus network but were not included in our Brain-GRFs list. Interestingly, one of these hubs, *GABPB1* encodes for a subunit of the hetero-tetrameric GABP consisting of two GABPA and two GABPB subunits (Batchelor et al., 1998). GABPA was recently found to bind human-specific binding sites and regulate gene expression of at least four genes (*ALDOA, HSPA8, TP73*, and *TMBIM6*) that have been associated with cognitive diseases such as autism, AZ, PD and other brain disorders (Perdomo-Sabogal et al., 2016). To 0 explore if more of these hubs might be associated with brain functions, we mined the (non-curated) data from DisGeNET (Piñero et al., 2015). We found that at least 12 of these hub GRFs may be connected with mental diseases and other neurological pathologies such as AZ (*DR1, ETS2, TFDP1, and TRIM13*), PD (*RUNX1T1*), SZ (*ZNF365*), developmental verbal dyspraxia (*ERC1*) and central neuroblastoma (*LMO3, PSMC5, TRIM13, TRIM24, ZMAT3*), among others. This suggests that with our method we have potentially identified novel candidates for being associated with important, if not essential, functions in the brain. We speculate that sequence and regulatory changes altering the regulatory activity or expression of these 36 hub GRFs could have medical relevance. It would thus be highly interesting to experimentally investigate their functions at brain level.

The structure and organization of the consensus network we are presenting here provides insights into regulatory circuits of the human frontal lobe. However, a yet unanswered question is how the network that we described for the human frontal lobe differs from the network of other brain regions, tissues or species. We expect that the relevant data for addressing this question will become available soon. We also expect that more GRFs will be discovered to be involved in brain functions. In future studies similar strategies as we presented here can then be implemented to enrich our knowledge about the molecular basis and regulatory networks underlying cognitive abilities.

## Author contributions

SB designed and executed research; AP contributed material; DG contributed analysis programs and visualizations; JQ contributed methods and designed research; KN designed research; All authors wrote and discussed the manuscript.

## Funding

This work was supported by a grant from Volkswagen Foundation within the initiative “Evolutionary Biology” awarded to KN and by a fellowship from the Departamento Administrativo de Ciencia, Tecnologia e Innovacion Colciencias from Colombia, calls Francisco Jose de Caldas 497/2009 awarded to AP. This work was funded by the Austrian Science Fund (FWF): M1619-N28 awarded to JQ.

### Conflict of interest statement

The authors declare that the research was conducted in the absence of any commercial or financial relationships that could be construed as a potential conflict of interest.
